# Spatiotemporal heterogeneity of core functional bacteria and their synergetic and competitive interactions in denitrifying sulfur conversion-assisted enhanced biological phosphorus removal

**DOI:** 10.1038/s41598-017-11448-x

**Published:** 2017-09-07

**Authors:** Yan Zhang, Mei Yu, Jianhua Guo, Di Wu, Zheng-Shuang Hua, Guang-Hao Chen, Hui Lu

**Affiliations:** 1 School of Environmental Science and Engineering, Sun Yat-sen University, Guangzhou, 510275 PR China; 2 Guangdong Provincial Key Laboratory of Environmental Pollution Control and Remediation Technology (Sun Yat-sen University), Guangzhou, 510275 PR China; 3 Advanced Water Management Centre (AWMC), The University of Queensland, St. Lucia, QLD 4072 Australia; 4 Department of Civil and Environmental Engineering, Chinese National Engineering Research Center for Control and Treatment of Heavy Metal Pollution (Hong Kong Branch), Fok Ying Tung Research Institute, The Hong Kong University of Science and Technology, Hong Kong, PR China; 5 State Key Laboratory of Biocontrol, Key Laboratory of Biodiversity Dynamics and Conservation of Guangdong Higher Education Institutes, College of Ecology and Evolution, Sun Yat-sen University, Guangzhou, PR China

## Abstract

Denitrifying sulfur conversion-assisted enhanced biological phosphorus removal (DS-EBPR) has recently been developed for simultaneously removing nitrogen and phosphorus from saline sewage with minimal sludge production. This novel process could potentially enable sustainable wastewater treatment. Yet, the core functional bacteria and their roles are unknown. Here, we used high-throughput 16S rRNA gene sequencing coupled with principal coordinates analysis and ANOVA with Tukey’s test to unravel the spatiotemporal heterogeneity of functional bacteria and their synergetic and competitive interactions. We did not find any obvious spatial heterogeneity within the bacterial population in different size-fractionated sludge samples, but the main functional bacteria varied significantly with operation time. *Thauera* was enriched (9.26~13.63%) as become the core functional genus in the DS-EBPR reactors and links denitrifying phosphorus removal to sulfide oxidation. The other two functional genera were sulfate-reducing *Desulfobacter* (4.31~12.85%) and nitrate-reducing and sulfide-oxidizing *Thiobacillus* (4.79~9.92%). These bacteria cooperated in the DS-EBPR process: *Desulfobacter* reduced sulfate to sulfide for utilization by *Thiobacillus*, *w*hile *Thauera* and *Thiobacillus* competed for nitrate and sulfide as well as *Thauera* and *Desulfobacter* competed for acetate. This study is the first to unravel the interactions among core functional bacteria in DS-EBPR, thus improving our understanding of how this removal process works.

## Introduction

For over 40 years, a process known as enhanced biological phosphorus removal (EBPR) has been successfully used to remove and recover phosphorus (P) in full-scale wastewater treatment plants. EBPR does not require chemical dosing for P precipitation and therefore produces less sludge than do conventional processes^1–3^. The functional bacteria in the EBPR system are referred to as polyphosphate-accumulating organisms (PAOs) and include *Candidatus* Accumulibacter phosphatis and *Tetrasphaera*-related bacteria^4–8^. PAOs compete extensively with glycogen-accumulating organisms (GAOs) in the EBPR system and are sensitive to both warm water temperatures (> 20°C)^9^ and high sulfate concentrations^10, 11^.

Wastewater contains sulfur (S) as a result of saltwater intrusion^12^, the application of sulfate coagulants in drinking water treatment^13^, brackish water supply systems^14^ and/or direct seawater toilet flushing^15^. In Hong Kong, seawater has been used for toilet flushing since 1958 with the installation of a dual water supply system to conserve fresh water resources and reduce energy consumption^16^. As a result, the sewage has a high ratio of sulfate-sulfur to chemical oxygen demand (COD) of ~0.4 mg S/mg COD^17^. This motivated a group of scientists and engineers to develop a completely new S-assisted biological wastewater treatment process called the sulfate reduction, autotrophic denitrification and nitrification integrated (SANI) process in the last decade for removing organics and nitrogen (N) with minimal sludge production^18–21^. To alleviate the eutrophication of receiving waters due to the presence of P in sewage, five years ago the same group extended the SANI process by introducing biological S-assisted denitrifying luxury P uptake so that besides organics and N, P could also be removed. The extended process is called denitrifying S cycle-assisted enhanced biological phosphorus removal (DS-EBPR)^11, 22^. In a tightly sealed sequencing batch reactor (SBR), polyphosphate (poly-P) is used to form poly-β-hydroxyalkanoates (PHAs) and convert polysulfide and/or elemental sulfur (poly-S) in anaerobic conditions. Nitrate is added into the SBR as soon as the acetate is exhausted, then the PHAs and poly-S is utilized for biological luxury P uptake in a denitrifying process. The process also can work even at temperatures as high as 30 °C^22^.

As DS-EBPR was only recently developed, few studies have been conducted and the ones available are mainly focused on process development, performance verification and evaluation. Some of these studies have touched on the microbial community governing this process and have even speculated about its underlying mechanism^11, 17, 22, 23^. For instance, one study using 454-pyrosequencing detected none of the conventional PAOs (i.e. *Candidatus* Accumulibacter and *Tetrasphaera*-related bacteria) in the microbial community of a DS-EBPR sludge sample cultivated under 30 ± 1°C; instead it found that the community was dominated by non-P-removing genera (83%) with unclassified genera making up the rest (17%)^22^. Another study using 16S rRNA gene pyrosequencing revealed that sulfate-reducing bacteria (SRB) and sulfide-oxidizing bacteria (SOB) were enriched at the genus level and highlighted the critical role of external sulfide addition in effectively restoring the DS-EBPR system from failure^17^. However, none of these studies has offered insights into the key functional bacteria governing the DS-EBPR process in carbon (C), N, P and S turnovers or the interactions between S-related and P-related bacteria in this complex microbial system, not to mention an in-depth understanding of bioprocess metabolism.

The overarching goal of this study is thus to unravel the spatiotemporal heterogeneity within the core functional bacterial population in different size-fractionated sludge samples during the long-term operation of DS-EBPR reactors and shed light on the synergetic and competitive interactions among these bacteria. To this end, three lab-scale DS-EBPR reactors were set up and operated for different periods of time to characterize the microbial community in the sludge samples. The characterization was performed on the Illumina MiSeq PE300 platform using high-throughput 16S rRNA gene sequencing. The temporal variation and spatial heterogeneity of the microbial community were investigated via principal coordinates analysis (PCoA) and ANOVA with Tukey’s test. The traits of functional bacteria and possible process metabolism and interactions during C, N, P and S conversions were revealed by combining the results of reactor operation and molecular bioinformation obtained from the DS-EBPR system.

## Results and Discussion

### Reactor performance

**Figure 1:**
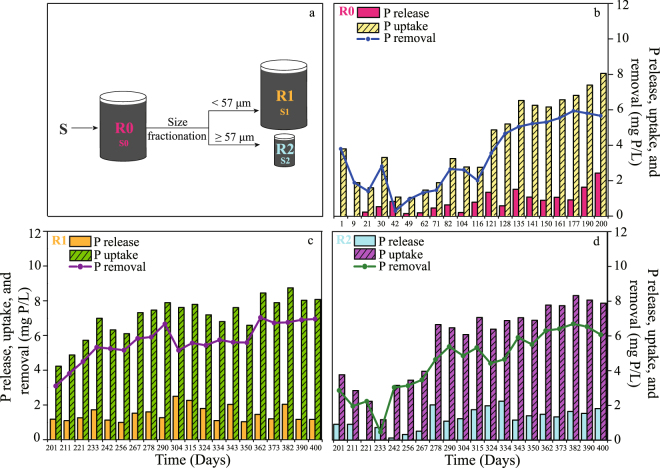
Experimental design and operational performance of the DS-EBPR process. (**a**) Experimental design and sampling. (**b**–**d**) P release, P uptake and P removal in each operation cycle of R0, R1 and R2.

**Figure 2:**
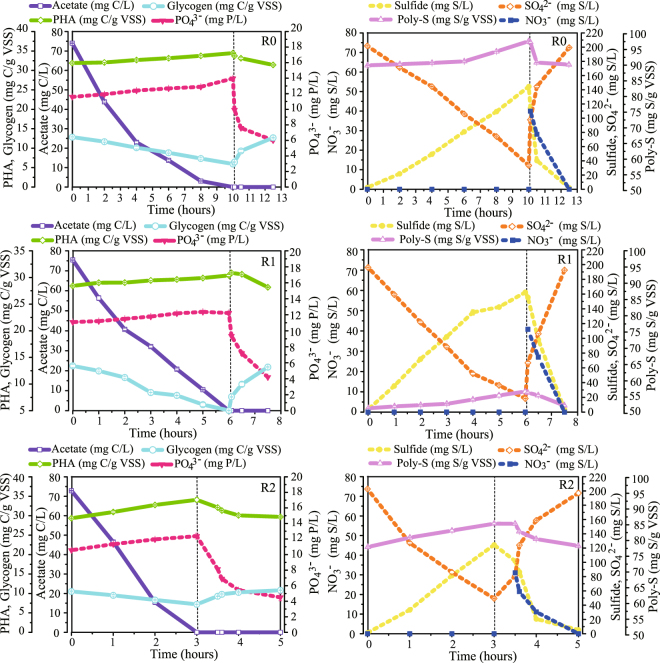
Typical cycle of the DS-EBPR process. (**a**–**c**) Typical cycle of R0, R1 and R2 on days 200, 400 and 400.

The operational performance, typical cycle measurements and performance of the three lab-scale reactors on the sampling dates are summarized in Figs 1 and 2 and Table 1. The P release, P uptake and P removal (calculated as P_uptake_ − P_release_) were measured to evaluate the EBPR performance and the results are shown in Fig. 1b,c and d respectively. The cycle time was significantly reduced from 24 and 14.5 h/cycle for reactor R0 (days 1~116 and days 117~200) to 9.5 h/cycle for R1 (days 201~400) and 7 h/cycle for R2 (days 201~400). The average P removal was determined to be 1.9 ± 0.3 mg P/(L·cycle) for R0 during days 1~116, 5.1 ± 0.2 mg P/(L·cycle) for R0 during days 117~200, 5.6 ± 1.0 mg P/(L·cycle) for R1 (days 201~400) and 4.5 ± 1.8 mg P/(L·cycle) for R2 (days 201~400). Therefore, R0 (after day 117), R1 and R2 were all able to remove P efficiently. The experimental stages and the initial concentrations of acetate, sulfate, phosphate and nitrate in the DS-EBPR SBRs are summarized in Supplementary Table S1. The representative cyclic performance of R0, R1 and R2 is shown in Fig. 2 (C and P conversions, as well as S and N conversions). The results demonstrate a clear association between S conversion and biological denitrifying P removal as reported in our previous study^11, 23^. Based on the experimental data in Table 1 such as the acetate uptake rate, the nitrate consumption rate, the sulfate reduction rate and the sulfide oxidation rate, the Spearman correlation was analyzed to investigate the significance of the operating conditions and reactor performance, and the results are shown in Supplementary Table S2. Phosphate removal was significantly and positively correlated with sulfide oxidation, initial acetate and nitrate (correlation coefficients > 0.7, *P* < 0.01). This might have been because the main P-removing bacteria could utilize acetate, nitrate and sulfide. However, phosphate removal was significantly and negatively correlated with initial sulfate (correlation coefficient > 0.7, *P* < 0.01). It was possible that the main P-removing bacteria could not utilize sulfate while other bacteria could and competed with the P-removing bacteria for the C source. These indicate that the microbes play a positive role in this complex ecosystem and cooperate to remove P. Hence, it was necessary to analyze the core functional microorganisms and their interactions.

**Table 1:** Operating conditions and performance of DS-EBPR SBRs (measured average value ± standard deviation, *n* = 3).

Operating conditions		R0	R1	R2
Reactors				
Day of sampling		Day 200	Day 400	Day 400
Temperature (°C)		22 ± 2	22 ± 2	22 ± 2
Working volume (L)		10.0	10.0	1.4
Sludge size (μm)		55.8 ± 0.4	63.2 ± 0.3	109.5 ± 0.5
VSS in reactor (g VSS/L)		3.3 ± 0.1	3.6 ± 0.1	4.0 ± 0.2
Sludge Retention Time (SRT) (d)		90	90	90
P release time (h/cycle)		10.0	6.0	3.5
P uptake time (h/cycle)		2.5	1.5	1.5
Cycle time (h/cycle)		14.5	9.5	7.0
Influent sewage (L/cycle)		5.0	5.0	0.7
**Reactor performance**				
Acetate	Initial (mg C/L)	74.0 ± 0.2	75.5 ± 0.2	72.9 ± 0.2
	End (mg C/L)	0.0	0.0	0.0
	Removal rate (%)	100	100	100
	Acetate-uptake rate (mg C/(g VSS·h))	1.6 ± 0.1	2.2 ± 0.1	2.6 ± 0.1
Nitrate	Initial (mg N/L)	40.0 ± 0.1	40.8 ± 0.1	31.0 ± 0.2
	End (mg N/L)	0.0	0.0	0.0
	Removal rate (%)	100	100	100
	Nitrate-consumption rate (mg N/(g VSS·h))	0.8 ± 0.1	1.2 ± 0.1	1.1 ± 0.1
Phosphate	Initial (mg P/L)	11.6 ± 0.1	11.1 ± 0.1	10.6 ± 0.1
	End (mg P/L)	6.0 ± 0.2	4.2 ± 0.1	4.5 ± 0.4
	Removal rate (%)	48.2	62.2	57.4
	Phosphate-removal rate (mg P/(g VSS·h))	0.1 ± 0.1	0.2 ± 0.1	0.2 ± 0.1
Sulfate	Initial (mg S/L)	201.8 ± 0.2	196.1 ± 0.2	202.7 ± 0.2
	End (mg S/L)	199.4 ± 0.4	192.4 ± 0.2	197.1 ± 3.3
	Sulfate-reduction rate (mg S/(g VSS·h))	3.5 ± 0.1	5.2 ± 0.1	5.5 ± 0.1
	Sulfide-oxidation rate (mg S/(g VSS·h))	3.0 ± 0.1	4.6 ± 0.1	4.3 ± 0.1

The average value of parameters was statistically calculated on day 200 from R0, as well as on day 400 from R1 and R2.

### Composition and spatiotemporal heterogeneity of the microbial community in DS-EBPR reactors

**Figure 3:**
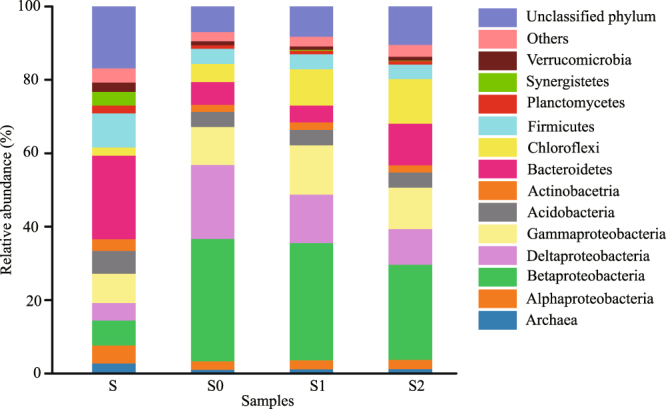
Relative abundance (%, *n* = 3) of the dominant microbial taxa in the inoculum (S) and the sludge samples (S0, S1 and S2).

An inoculum sludge sample S and sludge samples S0 (taken from R0 on day 200), S1 (taken from R1 on day 400) and S2 (taken from R2 on day 400) were analyzed on the Illumina MiSeq platform through the high-throughput sequencing of 16S rRNA to characterize the microbial community and determine its spatiotemporal heterogeneity in the DS-EBPR reactors. The operational taxonomic units (OTUs), as well as microbial diversity estimators such as ACE, Chao1, Shannon diversity index and Simpson index, are summarized in Supplementary Table S3. The data show that the microbial diversity was slightly reduced after a period (400 days) of S-assisted denitrifying P removal. The relative abundance of the dominant microbial taxa is shown in Fig. 3. Nine major bacterial phyla and archaea were detected in the three sludge samples, in which Proteobacteria were by far the most abundant phylum accounting for over half of the microbial community. This phylum also made up 24.4% of the microbial community in the inoculum sludge sample. Proteobacteria were also significantly enriched in our previous study^22^ and another study on the conventional EBPR process^24^. Betaproteobacteria accounted for 25.9~33.4% of the microbial community in the DS-EBPR reactors, much more so than other Proteobacteria and similar to the situation in the biological nutrient removal activated sludge system where Betaproteobacteria accounted for about 33% of the microbial community^25^. Chloroflexi and Bacteroidetes became more abundant the longer the reactors were operated. Both were enriched to a greater extent in the bigger biomass aggregates (S2 with average particle size of 109.5 ± 0.5 μm) than in the smaller ones (S1 with average particle size of 63.2 ± 0.3 μm) after sludge size fractionation, confirming their important role in biomass aggregation as reported in a study on the anaerobic sludge granulation of SRB for organics removal^26^. By comparing the sequences detected in the sludge samples (S0, S1 and S2) with BLAST against NCBI’s non-redundant database, we found that the three most abundant genera were *Thauera phenylacetica* (OTU1), *Desulfobacter postgatei* (OTU7, OTU578, OTU8276 and OTU10330) and *Thiobacillus thioparus* (OTU4) as shown in Supplementary Table S6. The phylogenetic trees in Supplementary Fig. S3 show the core functional lineages in the sludge samples and the common lineages.

**Figure 4:**
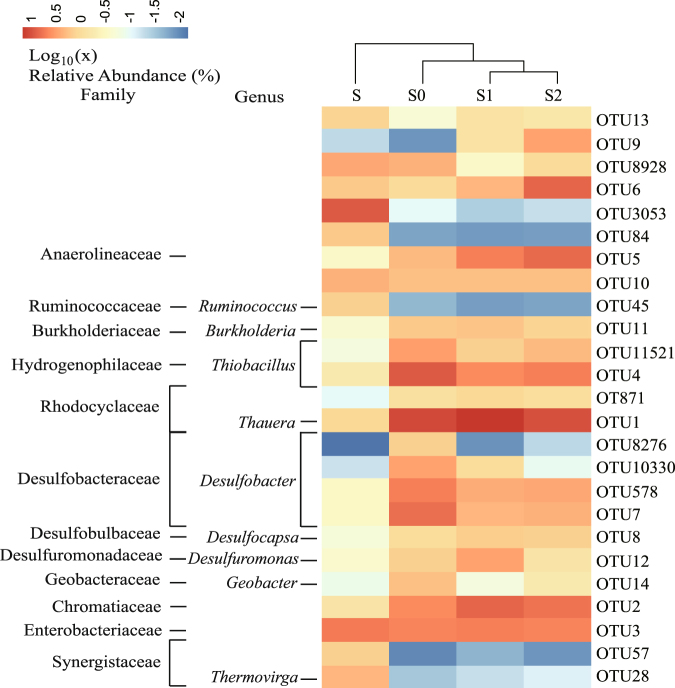
Heatmap of genera (relative abundance ≥ 1% in at least one sample). The color bar indicates the range of contribution of a genus to a sample, based on the color key (log_10_ scale) at the top. The family and genus names of the OTUs are shown on the left.

In order to investigate the temporal variation and spatial heterogeneity of the microbial community in the DS-EBPR reactors, Euclidean-based PCoA^27^ was conducted with 12 16S rRNA gene amplicons and 11 spatial dimensions. The first two principal coordinates (PC1 and PC2) explained 62.52% of the variance in microbial assemblages, in which PC1 alone explained 48.58%. Supplementary Fig. S2 shows three distinct clusters of data points: (1) those for the inoculum sludge sample are in yellow (S); (2) those for the sludge sample collected on day 200 are in blue (S0 from R0); and (3) those for the two sludge samples collected on day 400 are in red (S1 from R1) and green (S2 from R2). In general, the closer the clusters are together, the higher the similarity among microbial communities in the four sludge samples. This is also confirmed in Supplementary Table S4 with β-diversity (Bray-Curtis distance) metrics^28^. According to Supplementary Fig. S2 and Supplementary Table S4, the composition of the microbial community was primarily affected by the operating time, and no spatial heterogeneity existed between two of the size-fractionated sludge samples (S1 and S2). Similar conclusions could be drawn from the heatmap in Fig. 4 and the results of OTU abundance analysis in Supplementary Table S5. The relative abundances of *Thauera* (OTU1) and *Desulfuromonas* (OTU12) increased noticeably with operating time. On the contrary, the relative abundances of *Desulfobacter* (OTU7, OTU578, OTU8276 and OTU10330) and *Thiobacillus* (OTU4 and OTU11521) after 400 days of operation were much lower than those after just 200 days of operation.

### Key microorganisms involved in the metabolism of C, N, P and S

The relative abundance of the representive functional bacteria was at least ≥ 1% in each of S0, S1 and S2, implying that at least three bacteria (*Thauera*, *Thiobacillus* and *Desulfobacter*) significantly contributed to denitrifying S-assisted biological P removal. A total of nine functional traits for the three most abundant genera, i.e. *Thauera*, *Thiobacillus* and *Desulfobacter*, were coded from the closest related bacteria based on the phylogenetic functional traits of prokaryotes^29^. As indicated in Table 2, *Thauera phenylacetica* B4P (forming 9.24% and 12.66% of the microbial community in S0 and S1, respectively) was responsible not only for acetate uptake and PHA and glycogen formation but also for completing the glycolysis and tricarboxylic acid (TCA) cycle for acetate conversion^30^. However, *Desulfobacter postgatei* (4.89%) and *Desulfuromonas* (2.38%) were also detected in DS-EBPR reactor R1 after 200 days of operation. These bacteria utilize acetate as electron donors in anaerobic conditions for sulfate reduction. However, they were less abundant than *Thauera phenylacetica*, which was highly enriched in the DS-EBPR reactors after long-term operation. This was likely because *Thauera* can oxidize PHAs stored in cells through the TCA cycle to provide a proton-motive force for adenosine triphosphate (ATP) formation thereby accelerating the growth of cells^10, 31^.

**Table 2:** Functional trait analysis for three core functional genera regarding the metabolism of C, N, P and S in DS-EBPR reactors.

GENUS		*Thauera*		*Thiobacillus*	*Desulfobacter*
OTU ID		OTU1	OTU871	OTU4	OTU7 OTU578 OTU8276 OTU10330
Genome name		*Thauera phenylacetica* B4P		*﻿Thiobacillus thioparus﻿* DSM 505	*Desulfobacter postgatei* 2ac9
IMG Genome ID		2531839276		2515154076	2508501023
Competition-related traits^†^	Acetate uptake	+		−	+
	Formation/degradation of PHAs	+/+		−/−	−/−
	Formation/degradation of glycogen	+/+		+/+	+/+
	Full TCA cycle	+		+	+
	Denitrification	+		+	−
	Formation/degradation of Poly-P	+/+		+/+	+/+
	Sulfate reduction	−		−	+
	Sulfide oxidation	−		+	−
	Formation/degradation of Poly-S	+/−		+/+	−/−

^†^Traits are coded (+: trait present; −: trait absent) based on the genome of the closest genus.

For the N cycle, Table 2 shows that *Thauera phenylacetica* and *Thiobacillus thioparus* can trigger denitrification. *Thauera* and *Thiobacillus* coexist in autotrophic and heterotrophic denitrification processes, but their competition has never been documented because their relative abundances have never been analyzed statistically^32^. In our study, the relative abundance of *Thiobacillus* was reduced (from 9.92% to 4.79%) once the relative abundance of *Thauera* had increased from 9.98% to 13.63%, implying that the two competed for nitrate as electron acceptors when conditions in the DS-EBPR systems were anoxic.

For the P cycle, we speculate that *Thauera* is the core genus of bacteria responsible for P removal, based on the results of this study. *Thauera* has been reported to be not only a common denitrifier^33, 34^ but also a denitrifying P-removing agent^35^. In addition, we also found that *Thauera*, *Thiobacillus* and *Desulfobacter* could trigger the formation of poly-P (Table 2). However, *Thiobacillus* and *Desulfobacter* could not induce the formation of PHAs, which are needed for the formation of poly-P in anoxic conditions. PHAs are some of the most important intracellular polymers in P-removing bacteria. However, none of the conventional PAOs was detected in our DS-EBPR reactors. The main reason could be the strong presence of sulfide (approximately 124~162 mg S/L in R0, R1 and R2) at the end of the anaerobic phase in the DS-EBPR system. It has been reported that sulfide has a negative effect on normal PAO activity, and the effect is likely related to the concentration of undissociated H_2_S. Fifty percent inhibition of the maximum acetate uptake rate of PAOs has been observed at around 57 mg S/L^36^. Moreover, *Thauera* is the key enabler of the DS-EBPR process. Studies have shown that the energy production efficiency of PAOs with nitrate in the EBPR process could be up to 40% lower than that with oxygen^37^, leading to a 20–30% lower cell yield and achieving minimal biological sludge production^5^. The addition of nitrate in the DS-EBPR process could also reduce sludge production, besides reduction through S-cycle conversion^19^.

For the S cycle, *Desulfobacter postgatei*, which is known to be a sulfate-reducing bacterium^38^, was the main species that reduced sulfate to sulfide for utilization as electron donors in anaerobic conditions. The relative abundance of *Desulfobacter* fell sharply from 12.85 to 4.89%. In spite of this, it was still the most abundant among all of the S-reducing bacteria present. The anaerobic S-reducing bacterium *Desulfuromonas* ^39^ was also present in this system (0.57, 1.15, and 2.38% in R2, R0, and R1, respectively). However, its abundance increased from day 200 to day 400. Hence the two bacteria may be competitors, with *Desulfobacter* being less effective at utilizing C than *Desulfuromonas*. This result has been reported elsewhere^40^. Moreover, *Thiobacillus* can autotrophically oxidize inorganic S compounds through aerobic respiration or denitrification under anoxic conditions^32, 41, 42^. *Thiobacillus thioparus* is known as a nitrate-reducing sulfide-oxidizing bacterium^43^ and its traits are confirmed in Table 2. It is interesting to note that although the relative abundance of *Thiobacillus* decreased, the sulfide-oxidizing rates of R0, R1 and R2 were still approximately 3.0 ± 0.1, 4.6 ± 0.1 and 4.3 ± 0.1 mg S/(g VSS·h) respectively. The stable performance implies that *Thiobacillus* is not the only bacterium capable of oxidizing sulfide in the system. *Thauera* sp. may also oxidize sulfide^44, 45^. In addition, based on the genome, we also speculate that *Thauera* sp. can use poly-S as electron donors for autotrophic denitrification.

It should be mentioned that the microbial community in conventional EBPR is mainly dominated by conventional PAOs and supplanted by GAOs and the other major groups (e.g. Alphaproteobacterial, Planctomycete, and Flexibacter-CytophagaBacteroides^46^ or Xanthomonadales, Flavobacteriales, and Rhizobiales^4^). In contrast, none of the conventional PAOs were enriched in DS-EBPR. In fact, only S-related bacteria—*Thauera*, *Desulfobacter* and *Thiobacillus—*were enriched, which is not surprising as S cycle biotransformations are prevalent in the system.

We found a significantly positive correlation between phosphate removal and sulfide oxidation (correlation coefficient > 0.7, *P* < 0.01) (Supplementary Table S2). Thus *Thauera* is likely to be the key functional bacterium for denitrifying P removal and to be associated with sulfide oxidation in the DS-EBPR system. Moreover, the purple and green S bacteria (i.e. OTU2 and OTU11) might play a role in the conversion of poly-S. These bacteria can oxidize sulfide to poly-S using CO_2_ as electron acceptors^47^. The poly-S could then be oxidized to sulfate^48^. However, in this study we could not confirm the exact pathway of poly-S formation. Metagenomic and metatranscriptomic studies are needed to obtain more solid evidence and insights.

### Competitive and cooperative interactions among core functional microorganisms in the DS-EBPR system

The Spearman correlation between operating conditions and reactor performance was calculated (see Supplementary Table S2). Phosphate removal was significantly and positively correlated with initial acetate and nitrate levels (correlation coefficients > 0.7, *P* < 0.01) because the two were utilized by *Thauera*. However, it was significantly and negatively correlated with the initial sulfate level (correlation coefficient > 0.7, *P* < 0.01). SRB might compete with *Thauera* for C. They might grow more strongly when more sulfate is present, which would explain the negative correlation between phosphate removal and the initial sulfate level. However, the lack of sulfate initially was not necessarily beneficial for phosphate removal. The net P removal decreases when sulfate reduction weakens^22^. In addition, this study shows that the presence of SRB benefits the reduction of sulfate and the generation of sulfide, the latter of which is then utilized by SOB in DS-EBPR. Meanwhile, phosphate removal was significantly and positively correlated with sulfide oxidation (correlation coefficient > 0.7, *P* < 0.01). The Spearman correlation further confirms that *Thauera* was the denitrifying polyphosphate-accumulating organism (DPAO) and was likely associated with sulfide oxidation. Thus cooperation and competition were both observed in the DS-EBPR system.

**Figure 5:**
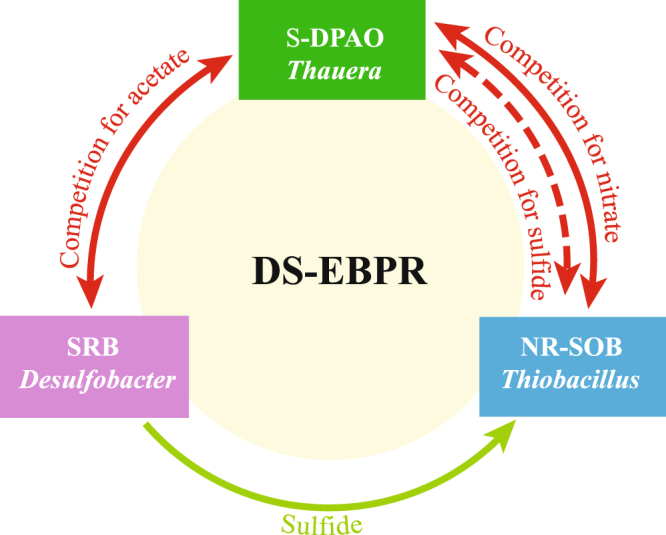
Possible competition and cooperation among bacteria in DS-EBPR. S-DPAO stands for denitrifying polyphosphate-accumulating organism and is associated with sulfide oxidation; SRB represents sulfate-reducing bacteria; NR-SOB means nitrate-reducing and sulfide-oxidizing bacteria. Solid lines represent competition or cooperation, whereas the dotted line represents competition for sulfide.

Based on this and our previous studies^11, 17, 22, 23^, we propose for the first time that competitive and cooperative metabolic interactions (Fig. 5) occur within the microbial community in the DS-EBPR system. *Thauera* is likely a major contributor to P removal. Equation (1) describes the generation and degradation of poly-P^23^. Other PAO-related bacteria may have been present but went undetected because their concentrations were too low and the functional traits of their genomes are unknown. *Thauera* and SRB (mainly *Desulfobacter*) compete with each other for acetate. *Desulfobacter* oxidizes acetate and reduces sulfate to sulfide under anaerobic conditions, as described in equation (2)^49^. Meanwhile, SOB utilize sulfide as electron donors in the DS-EBPR system. Studies have demonstrated syntrophic growth between SRB and SOB, such that a positive-feedback S cycle is established between the reductive and oxidative processes of these two kinds of bacteria^50^. Moreover, *Thiobacilus* can also stimulate further sulfide oxidation with the help of nitrate^42^, as described in equation (3)^51^. Furthermore, *Thiobacillus* may compete with *Thauera* for nitrate as electron acceptors and sulfide as electron donors in the DS-EBPR system. Competition existed among bacteria, but on the whole the core functional bacteria related to C, N, P and S interacted syntrophically in the DS-EBPR system. This work demonstrates conceptual metabolic interactions. The results help improve our understanding of the DS-EBPR system, allowing us to incorporate insights derived from laboratory experiments into a framework that can potentially be used to assess risk and design optimal schemes for treating wastewater.

1 $${\rm{ATP}}+{{\rm{H}}}_{3}{{\rm{PO}}}_{4}\leftrightarrow {{\rm{HPO}}}_{3}({\rm{poly}}-{\rm{P}})+{{\rm{H}}}_{2}{\rm{O}}$$

2 $${{\rm{Acetate}}}^{-}+{{{\rm{SO}}}_{4}}^{2-}\to {{\rm{HS}}}^{-}+2\,{{{\rm{HCO}}}_{3}}^{-}$$

3 $$5\,{{\rm{HS}}}^{-}+8\,{{{\rm{NO}}}_{3}}^{-}+3\,{{\rm{H}}}^{+}\to 5\,{{{\rm{SO}}}_{4}}^{2-}+4\,{{\rm{N}}}_{2}\uparrow +4\,{{\rm{H}}}_{2}{\rm{O}}$$

## Methods

### Reactor setup, seeding inoculum and operation

This study used three separate tightly-sealed SBRs (R0, R1 and R2) for denitrifying S-assisted P removal as shown in Supplementary Table S1 of supplementary information (SI). These reactors were operated under similar alternating anaerobic/anoxic conditions according to the following operating steps: (1) the SBRs were fed with synthetic sewage for 20 min with the influent volumes shown in Table 1 (sewage characteristics are shown in SI); (2) P release, i.e. reaction phase R, occurred in anaerobic conditions for different periods of time as shown in Table 1; (3) the sewage was spiked with 100 mL of nitrate solution with an average concentration of 40 mg N/L for R0 and R1 and 31 mg N/L for R2; (4) P uptake, i.e. reaction phase U, occurred in anoxic conditions for different periods of time as shown Table 1; and (5) settling and decanting lasted 80 and 20 min, respectively.

Reactor R0 with a working volume of 10 L was initially inoculated with concentrated flocculent activated sludge (having an average particle size of 55.8 ± 0.4 μm) obtained from the secondary sedimentation tank of the Sha Tin Sewage Treatment Works, the largest saline sewage treatment plant in Hong Kong. This reactor was operated according to the five steps described above with mechanical stirring at 200 rpm at 22 ± 2 °C for 200 days to cultivate functional microorganisms and achieve denitrifying S-assisted P removal. In order to investigate the spatial heterogeneity of functional microorganisms in the DS-EBPR reactors, 10 L of activated sludge were removed from R0 on day 200 and immediately filtered with a mesh screen having a pore size of 57 μm for size fractionation following the protocol used in our previous study^52^. We thus obtained 0.2 L of concentrated sludge with an average particle size exceeding 57 μm which we then transferred to a smaller reactor R2 (with a working volume of 1.4 L). The initial sludge concentration was 3.8 ± 0.2 mg/L mixed liquor volatile suspended solids (MLVSS). Gentle stirring was applied at 150 rpm at 22 ± 2 °C to promote biomass aggregation. Sludge with an average particle size of less than 57 μm after size fractionation was transferred to another reactor R1 (with a working volume of 10 L). In this case the initial sludge concentration was 2.9 ± 0.2 mg/L MLVSS and stirring was applied at 200 rpm at 22 ± 2 °C to prevent biomass aggregation. Figure 1a illustrates the setup. Images of sludge samples S1 and S2 were taken using a field emission scanning electron microscope and are shown in Supplementary Fig. S1.

### Sludge sampling, DNA extraction, polymerase chain reaction (PCR) amplification, and Illumina MiSeq sequencing

In order to investigate the spatial and temporal changes in the bacterial population in the DS-EBPR reactors, an inoculum sludge sample S was taken on day 0 and three activated sludge samples were taken on day 200 (S0 from reactor R0 after cultivation for 200 days) and day 400 (S1 from R1 and S2 from R2 after size fractionation and cultivation for another 200 days) (as shown in Fig. 1a). The DNA in each sludge sample was extracted immediately after sludge sampling via the following steps^28^: (1) 2 mL of thoroughly mixed sludge sample were centrifuged at 4 °C for 10 min at 10,000 g in a high-speed centrifuge (HERMLE Labortechnik GmbH, Germany); (2) after pouring out the suspension, 200 mg of sludge were transferred to a 2 mL DNA LoBind tube (Eppendorf, Germany) for DNA extraction; and (3) the community DNA of microbes was extracted using the FastDNA Spin Kit (Qbiogene, Carlsbad, CA) according to the manufacturer’s instructions.

The universal primer set F515/R806 was used to amplify the V4 hypervariable region of the 16S rRNA gene. This region covered nearly all bacterial and archaeal taxa^53^. The DNA samples were amplified in 50 μL of reaction mixtures. Amplification was performed with the GeneAmp PCR System 9700 (ABI, US) according to the following steps: denaturing at 94 °C for 5 min, followed by 31 cycles of denaturing at 94 °C for 30 s, annealing at 52 °C for 30 s, extension at 72 °C for 45 s, and final extension at 72 °C for 10 min. Replicate amplicons were pooled for purification with the QIAquick Gel Extraction Kit (Qiagen, Chatsworth, CA). The integrity of the DNA and PCR amplification was verified using agarose gel electrophoresis, and concentration and purity were determined using the NanoDrop 2000 spectrophotometer (Thermo Fisher Scientific, US). Adaptor-appended fragments were sequenced on the Illumina MiSeq PE300 platform. The sequences were deposited in the European Molecular Biology Laboratory (EMBL) sequence read archive with accession number ERR1778916.

### Bioinformatic analysis and data availability

The raw paired-end reads with a Phred quality score of less than 20 at the 3’ end were trimmed using a custom Perl script. Contigs were then assembled using FLASH^54^ for each read pair. Chimeric contigs were identified and removed using USEARCH (version 8.1.1861). Then the quality sequences were processed using the pipelines of Mothur^55^ (version 1.29) and QIIME^56^. Briefly, we clustered all of the contigs at the 97% similarity level to obtain OTUs for all samples. Taxonomic information was determined using the Ribosomal Database Project (RDP) Classifier^57^ (version 2.6) with a minimum confidence of 80%. The relative abundance of each individual OTU was computed as the ratio of the sequence number of that OTU to the total number of sequences assigned to that sample. To avoid bias caused by sequencing depth, we sampled the microbial community by taking the smallest sample size of 10,000 sequences to calculate the metrics of α-diversity (mainly the ACE, Chao1, Shannon and Simpson indexes) and *β*-diversity (e.g., Bray-Curtis, weighted unifrac and unweighted unifrac). Neighbor-joining-based phylogenetic trees of core OTUs and their closely related species were generated using MEGA^58^ (version 5.2). The Euclidean-based PCoA was used to show the different assemblages between the DS-EBPR sludge samples and the seeding sludge. Independent sample t-tests based on Bray-Curtis distances (*P* < 0.05), Spearman correlation (two-tailed) and one-way ANOVA followed by Tukey and Tamhane’s T2 post-hoc test (*P* < 0.05) were performed in SPSS 18.0. A heatmap was generated using the pheatmap package in the statistical program R. Sequences were compared using BLAST against NCBI’s non-redundant database (Supplementary Table S6). Bacterial functional traits are detailed in SI.

### Chemical analysis

Mixed liquor suspended solids, MLVSS and sulfide were determined according to Standard Methods^59^. Key anions in the bulk liquid, including acetate, nitrate, nitrite, phosphate, thiosulfate, sulfite and sulfate, were determined with an ion chromatograph equipped with an IC-AS23 analytical column (DIONEX ICS-900, US). The concentration of poly-P (in mg P/L) was calculated from the changes in the bulk liquid phosphate concentration^22^. Glycogen and poly-S were detected following previous methods^11^. PHAs were determined with a high-performance liquid chromatograph (HPLC, Dionex Ultimate 3000, US) following the protocol described in SI. The sludge size was measured using the Mastersizer 3000 laser diffraction floc size analyzer (Malvern Instruments Ltd., UK). A scanning electron microscope was used to take pictures of sludge samples prepared according to procedures detailed in Kloep *et al*.^60^.

### Data availability

The datasets generated in the current study are available from the corresponding author on request.

## Electronic supplementary material

Supplementary information
