# Supplementary material for: Spatiotemporal heterogeneity of core functional bacteria and their synergetic and competitive interactions in denitrifying sulfur conversion-assisted enhanced biological phosphorus removal
Source: Sci Rep. 2017 Sep 7;7:10927. doi: 10.1038/s41598-017-11448-x (PMC5589776; doi:10.1038/s41598-017-11448-x)
Supplement: Supplementary file 1 — Supplementary information [file 41598_2017_11448_MOESM1_ESM.pdf]

Spatiotemporal heterogeneity of core functional bacteria and their synergetic and competitive interactions in denitrifying sulfur conversion-assisted enhanced biological phosphorus removal

Yan Zhang[1,2], Mei Yu[1,2], Jianhua Guo[3], Di Wu[4*], Zheng-Shuang Hua[5], Guang-Hao Chen[4] and Hui Lu[1,2*]

[1] School of Environmental Science and Engineering, Sun Yat-sen University, Guangzhou 510275, PR China.

[2] Guangdong Provincial Key Laboratory of Environmental Pollution Control and Remediation Technology (Sun Yat-sen University), Guangzhou 510275, PR China.

[3] Advanced Water Management Centre (AWMC), The University of Queensland, St. Lucia, QLD 4072, Australia.

[4] Department of Civil and Environmental Engineering, Chinese National Engineering Research Center for Control and Treatment of Heavy Metal Pollution (Hong Kong Branch), Fok Ying Tung Research Institute, The Hong Kong University of Science and Technology, Hong Kong, PR China.

[5] State Key Laboratory of Biocontrol, Key Laboratory of Biodiversity Dynamics and Conservation of Guangdong Higher Education Institutes, College of Ecology and Evolution, Sun Yat-sen University, Guangzhou, PR China.

[*]**Corresponding authors:** lvhui3@mail.sysu.edu.cn; cewudi@ust.hk

Supplementary Information

Supplementary Table S1

Supplementary Table S2

Supplementary Table S3

Supplementary Table S4

Supplementary Table S5

Supplementary Table S6

Supplementary Figure S1

Supplementary Figure S2

Supplementary Figure S3

Influent characteristics

Synthetic wastewater contained 400 mg/L COD and 20 mg P/L. It was prepared from 0.5210 g/L CH_3COONa as the sole carbon source, 0.8880 g/L Na_2SO_4, 0.0670 g/L K_2HPO_4 and 0.0350 g/L KH_2PO_4. Appropriate macro-minerals were added to the feed by adding 0.0390 g/L $MgCl_2$, 0.0520 g/L $CaCl_2$, 0.0946 g/L NH_4Cl and 0.0100 g/L EDTA, and 2.0 ml/L trace mineral solution (0.0200 g/L $FeCl_3{\cdot}6H_2O$, 0.0020 g/L H_3BO_3, 0.0005g/L $CuSO_4$, 0.0008 g/L KI, 0.0025 g/L $MnSO_4{\cdot}H_2O$, 0.0015 g/L $ZnSO_4{\cdot}7H_2O$, 0.0020 g/L $CoCl_2{\cdot}6H_2O$, without $(NH_4)_6Mo_7O_{24}{\cdot}4H_2O$ and $C_6H_5Na_3O_7{\cdot}2H_2O$), nitrate prepared from sodium nitrate was the electron acceptor[1]. The cyclic operation of the SBR, including: (i) feeding of synthetic wastewater, (ii) P-release phase, (iii) addition of nitrate, (iv) P-uptake phase, (v) settling over night, and (vi) decantation of supernatant (half of total volume).

Bacterial functional traits

The genomic information of the bacteria nearest to the core OTUs was available, we downloaded protein coding genes connected to KEGG (Kyoto Encyclopedia of Genes and Genomes) Orthology (KO) from the Integrated Microbial Genomes (IMG) database[2]. We specifically considered nine traits conferring performance of C, N, P and S conversions in the DS-EBPR process. We coded: (i) acetate consumption, that allows growth on anaerobic conditions, due to the possession of *act*, *acs*, *ack* and *pta* genes . They clearly have the potential to take up and utilize acetate[3]; (ii) formation of PHA and glycogen, which serve as carbon and energy storage materials that are formed in response to nutritional imbalances, the key genes are the *phaC* and *gpi* (coding for polyhydroxyalkanoate synthase and glucose 6-phosphate isomerase)[4]; (iii) denitrification, involvement in any step from the reduction of nitrate to the production of molecular nitrogen, either due to the possession of *nar* and/or *nap*, *nir*, *nor* and *nos* genes (respectively, coding for nitrate, nitrite, nitric oxide and nitrous oxide reductases)[5]; (iv) formation and degradation of poly-phosphate (poly-P), due to the possession of *ppk1* and *ppx* gene (coding for polyphosphate kinase and exopolyphosphatase)[6]; (v) sulfate reduction and sulfide oxidation, that is the dissimilatory sulfate reduction and oxidation by the *sat*, *apr* and *dsr* (coding for sulfate adenylyltransferase, adenylylsulfate reductase, dissimilatory sulfite reductase, respectively)[7]; (vi) formation of poly-S possibly serving as electron and energy storage material like PHA and glycogen[8] the key gene is the *sqr* (coding for sulfide

quinone oxidoreductase)[9]. The poly-S are able to transform to sulfate via enzymes encoded by the *dsr* gene[10]. Coding of each trait was based on the information of functional genes involved in the downloading genera (+: trait existed; −: trait did not exist and an extensive review of literature, as shown in Table 2).

PHA measurement

The PHA was determined with high performance liquid chromatography (HPLC). Approximately 15 mg weighted pellets were added to 10 mL glass digestion tube, then added 0.5 mL 98% H_2SO_4 and 0.5 mL 100% methanol in turn. Six standards were composed of 0~2 mg of R-3-hydroxybutyric (3HB) and R-3-hydroxyvaleric acid (3HV) copolymer (12% HV, Sigma-Aldrich). The samples and standards were all digested for 2 h at 105°C to form methyl crotonate, and then cooled down to room temperature. 9 mL of ultrapure water was added and mixed, and then filtrated with a 0.22 μm Jin Teng nylon syringe filter. The methyl crotonate was determined by HPLC (Dionex Ultimate 3000) equipped with a C18 column (150 mm × 2.1 μm × 3 μm) (Thermofisher)[11].

Phylogenetic tree

A phylogenetic tree for the core functional lineages from the DS-EBPR and some of reported functional bacteria was built. As shown in the phylogenetic trees (Fig. S3 a, b and c), the traditional PAOs, *Rhodocyclus* sp. clade IA, *Candidatus* Accumulibacter phosphatis clade IIA, *Tetrasphaera* which have been considered to dominate phosphate-removing sludge populations. The nearest to OTU1 and OUT871 belong to the *Thauera,* which also belongs to the DPAO. The OTU4 and OTU11521, sequenced as *Thiobacillus thioparus* and *Thiobacillus* sp., belong to sulfide-oxidizing bacterial lineages. The other major OTUs (i.e. OTU7, OTU578, OTU8276 and OTU10330 sequenced as *Desulfobacter postgatei*, OTU8 sequences as *Desulfocapsa sulfoexigens*, and OTU12 sequenced as *Desulfuromonas acetexigens*, respectively.) belong to sulfate-reducing bacterial or elemental sulfur lineages. The phylogenetic trees indicate that the sequencing classification is reliable and give a look at their evolutionary relationships between core functional bacteria and related functional bacteria reported.

Reactors		R0		R1	R2
Stages		Stage 1	Stage 2	Stage 2	Stage 2
Day		1 ~ 116	117 ~ 200	201 ~ 400	201 ~ 400
SRT[a] (day)		100	90	90	90
Cycle time (h/cycle)		24.0	14.5	9.5	7.0
VSS concentration (g VSS/L)		3.1 ± 0.4	3.2 ± 0.2	3.5 ± 0.2	4.1 ± 0.1
Initial concentration	Acetate[b] (mg C/L)	75.0 ± 6.2	73.9 ± 4.5	74.6 ± 4.9	73.3 ± 5.9
	Sulfate[b] (mg S/L)	198.7 ± 11.3	201.2 ± 5.1	199.0 ± 9.9	202.6 ± 8.0
	Phosphate[b] (mg P/L)	11.1 ± 1.1	11.5 ± 1.1	11.2 ± 0.8	10.7 ± 1.0
	Nitrate[b] (mg N/L)	30.7 ± 7.0	31.6 ± 7.3	32.6 ± 5.3	30.6 ± 2.4

Supplementary Table S1. Experimental stages and initial concentrations of acetate, sulfate, phosphate and nitrate in DS-EBPR SBRs (measured average value ± standard deviation). [a] The sludge retention time (SRT) was calculated by dividing the total reactor biomass by the average daily biomass wastage via effluent and sampling. [b] The initial concentration of acetate, sulfate and phosphate were measured at the beginning of anaerobic conditions and nitrate of anoxic conditions.

Operating condition and reactor performance		Initial Acetate (mg C/L)	Initial Nitrate (mg N/L)	Initial Phosphate (mg P/L)	Initial Sulfate (mg S/L)	Acetate-uptake (mg C/g VSS)	Nitrate-consumption (mg N/g VSS)	Phosphate-removal (mg P/g VSS)	Sulfate-reduction (mg S/g VSS)	Ssulfide-oxidation (mg S/g VSS)
Initial acetate (mg C/L)	C. C.	1	.817**	0.417	-.817**	0.550	0.367	.800**	0.350	.800**
	Sig.	.	0.007	0.265	0.007	0.125	0.332	0.010	0.356	0.010
Initial nitrate (mg N/L)	C. C.	.817**	1	0.500	-.967**	0.367	0.550	.983**	0.533	.983**
	Sig.	0.007	.	0.170	0	0.332	0.125	0	0.139	0
Initial phosphate (mg P/L)	C. C.	0.417	0.500	1	-0.467	.867**	.950**	0.483	.933**	0.483
	Sig.	0.265	0.170	.	0.205	0.002	0	0.187	0	0.187
Initial sulfate (mg S/L)	C. C.	-.817**	-.967**	-0.467	1	-0.367	-0.517	-.983**	-0.533	-.983**
	Sig.	0.007	0	0.205	.	0.332	0.154	0	0.139	0
Acetate-uptake	C. C.	0.550	0.367	.867**	-0.367	1	.817**	0.350	.800**	0.350
(mg C/g VSS)	Sig.	0.125	0.332	0.002	0.332	.	0.007	0.356	0.010	0.356
Nitrate-consumption	C. C.	0.367	0.550	.950**	-0.517	.817**	1	0.533	.983**	0.533
(mg N/g VSS)	Sig.	0.332	0.125	0	0.154	0.007	.	0.139	0	0.139
Phosphate-removal	C. C.	.800**	.983**	0.483	-.983**	0.350	0.533	1	0.545	.986**
(mg P/g VSS)	Sig.	0.010	0	0.187	0	0.356	0.139	.	0.067	0
Sulfate-reduction	C. C.	0.350	0.533	.933**	-0.533	.800**	.983**	0.545	1	0.531
(mg S/g VSS)	Sig.	0.356	0.139	0	0.139	0.010	0	0.067	.	0.075
Sulfide-oxidation	C. C.	.800**	.983**	0.483	-.983**	0.350	0.533	.986**	0.531	1
(mg S/g VSS)	Sig.	0.010	0	0.187	0	0.356	0.139	0	0.075	.

Supplementary Table S2. Spearman correlations computed using the operating condition and reactor performance (per cycle) of DS-EBPR SBRs. C. C. means Correlation Coefficient, sig. means significant, * $P < 0.05$ level (2-tailed), ** $P < 0.01$ level (2-tailed).

	No. of OTUs	ACE	Chao1	Shannon	Simpson
S	1583 ± 241	2731 ± 317	2357 ± 325	8.90 ± 0.12	0.99 ± 0
S0	1604 ± 224	2502 ± 166	2228 ± 126	7.49 ± 0.02	0.98 ± 0
S1	1771 ± 353	2489 ± 160	2204 ± 178	7.53 ± 0.10	0.97 ± 0
S2	2121 ± 244	2713 ± 66	2418 ± 76	7.58 ± 0.08	0.98 ± 0

Supplementary Table S3. Summary of operational taxonomic units (OTUs) and microbial diversity estimates (measured average value ± standard deviation, *n* = 3).

	P value
Distances among S cluster vs. that between S cluster and S0 cluster	< 0.001
Distances among S cluster vs. that between S cluster and S1 cluster	< 0.001
Distances among S0 cluster vs. that between S0 cluster and S1 cluster	0.002
Distances among S1 cluster vs. that between S1 cluster and S2 cluster	0.028

Supplementary Table S4. Differences between Bray_Curtis distances among the seeding sludge (S) and DS-EBPR SBRs samples (S0, S1 and S2) and that between them using the independent samples t-test.

	S	S0	S1	S2
OTU13	1.03 ± 0.31^{a}	0.18 ± 0.05^{bc}	0.65 ± 0.09^{ab}	0.53 ± 0.06^{bc}
OTU9	0.05 ± 0.03^{b}	0.01 ± 0.01^{b}	0.67 ± 0.20^{b}	2.48 ± 0.83^{a}
OTU8928	2.20 ± 0.56^{a}	1.93 ± 0.38^{a}	0.24 ± 0.13^{b}	0.91 ± 0.19^{b}
OTU6	1.37 ± 0.23^{cd}	0.89 ± 0.14^{d}	1.85 ± 0.05^{c}	6.14 ± 0.48^{a}
OTU3053	7.22 ± 1.31^{a}	0.09 ± 0.04^{b}	0.03 ± 0.02^{b}	0.05 ± 0.04^{b}
OTU84	1.35 ± 0.36^{a}	0.01 ± 0.02^{b}	0.01 ± 0.00^{b}	0.01 ± 0.02^{b}
OTU5	0.23 ± 0.04^{e}	1.7 ± 0.21^{d}	4.09 ± 0.33^{b}	5.59 ± 0.30^{a}
OTU10	1.90 ± 0.23^{a}	1.57 ± 0.17^{a}	1.55 ± 0.05^{a}	1.52 ± 0.17^{a}
OTU45	1.13 ± 0.21^{a}	0.02 ± 0.03^{b}	0.01 ± 0.01^{b}	0.01 ± 0.02^{b}
OTU11	0.19 ± 0.05^{c}	1.29 ± 0.16^{ab}	1.43 ± 0.13^{ab}	1.03 ± 0.13^{b}
OTU11521	0.14 ± 0.02^{d}	2.59 ± 0.26^{a}	1.15 ± 0.07^{c}	1.68 ± 0.21^{b}
OTU4	0.45 ± 0.06^{d}	7.33 ± 0.72^{a}	3.64 ± 0.12^{b}	4.08 ± 0.44^{b}
OTU871	0.09 ± 0.03^{c}	0.74 ± 0.13^{b}	0.97 ± 0.13^{ab}	0.75 ± 0.02^{b}
OTU1	0.97 ± 0.15^{c}	9.24 ± 0.35^{b}	12.66 ± 1.08^{a}	8.51 ± 0.13^{b}
OTU8276	0.01 ± 0.01^{b}	1.15 ± 0.09^{a}	0.01 ± 0.01^{b}	0.04 ± 0.01^{b}
OTU10330	0.05 ± 0.04^{c}	2.53 ± 0.18^{a}	0.86 ± 0.11^{b}	0.1 ± 0.10^{c}
OTU578	0.24 ± 0.07^{c}	4.09 ± 0.88^{a}	2.16 ± 0.10^{b}	2.22 ± 0.84^{b}
OTU7	0.24 ± 0.05^{c}	5.08 ± 0.38^{a}	1.86 ± 0.43^{b}	1.95 ± 0.56^{b}
OTU8	0.17 ± 0.09^{c}	0.81 ± 0.15^{b}	1.25 ± 0.12^{b}	1.15 ± 0.13^{b}
OTU12	0.22 ± 0.09^{c}	1.15 ± 0.20^{b}	2.38 ± 0.09^{a}	0.57 ± 0.15^{c}
OTU14	0.12 ± 0.05^{d}	1.54 ± 0.03^{a}	0.14 ± 0.01^{d}	0.44 ± 0.01^{b}
OTU2	0.62 ± 0.16^{d}	3.61 ± 0.26^{c}	6.16 ± 0.89^{ab}	4.91 ± 0.47^{bc}
OTU3	4.38 ± 1.37^{a}	3.94 ± 0.19^{a}	4.28 ± 0.12^{a}	3.96 ± 0.11^{a}
OTU57	1.12 ± 0.14^{a}	0.01 ± 0.02^{b}	0.02 ± 0.01^{b}	0.01 ± 0.01^{b}
OTU28	1.87 ± 0.10^{a}	0.03 ± 0.02^{b}	0.05 ± 0.02^{b}	0.07 ± 0.02^{b}

Supplementary Table S5. Relative abundance (≥ 1% in at least one sample) among seeding sludge (S) and those representing the DS-EBPR SBRs samples (S0, S1 and S2) by one-way ANOVA Tukey post-hoc test (*P* < 0.05) (measured average value ± standard deviation, *n* = 3). [a, b and c] The measured average value is significant at the 0.05 level.

[illegible] ID	Consensus Lineage	Closest organism	Similarity
1	Proteobacteria; Betaproteobacteria; Rhodocyclales; Rhodocyclaceae; *Thauera*	*Thauera phenylacetica*	98%
2	Proteobacteria; Gammaproteobacteria; Chromatiales; Chromatiaceae	Chromatiaceae bacterium	100%
3	Proteobacteria; Gammaproteobacteria; Enterobacteriales; Enterobacteriaceae; *Enterobacter*	*Enterobacter cloacae*	100%
4	Proteobacteria; Betaproteobacteria; Hydrogenophilales; Hydrogenophilaceae; *Thiobacillus*	*Thiobacillus thioparus*	98%
5	Chloroflexi; Anaerolineae; Anaerolineales; Anaerolineaceae; *Bellilinea*	*Bellilinea* sp.	99%
6	Bacteroidetes; Bacteroidetes; Bacteroidales; Bacteroidaceae; *Cytophaga*	*Cytophaga* sp.	99%
7	Proteobacteria; Deltaproteobacteria; Desulfobacterales; Desulfobacteraceae; *Desulfobacter*	*Desulfobacter postgatei*	100%
8	Proteobacteria; Deltaproteobacteria; Desulfobacterales; Desulfobulbaceae	Desulfobulbaceae bacterium	98%
9	Chloroflexi; Dehalococcoidia; *Dehalogenimonas*;	*Dehalogenimonas* sp.	96%
10	Firmicutes; Bacilli; Bacillales; Bacillaceae; *Bacillus*	*Bacillus* sp.	100%
11	Proteobacteria; Betaproteobacteria; Burkholderiales; Burkholderiaceae; *Burkholderia*	*Burkholderia cepacia*	99%
12	Proteobacteria; Deltaproteobacteria; Desulfuromonadales; Desulfuromonadaceae; *Desulfuromonas*	*Desulfuromonas* sp.	100%
13	Deferribacteres; Deferribacteres; Deferribacterales; Deferribacteraceae; *Deferribacter*	*Deferribacter* sp.	100%
14	Proteobacteria; Deltaproteobacteria; Desulfuromonadales; Geobacteraceae; *Geobacter*	*Geobacter lovleyi*	99%
578	Proteobacteria; Deltaproteobacteria; Desulfobacterales; Desulfobacteraceae; *Desulfobacter*	*Desulfobacter postgatei*	99%
871	Proteobacteria; Betaproteobacteria; Rhodocyclales; Rhodocyclaceae; *Thauera*	*Thauera phenylacetica*	97%
8276	Proteobacteria; Deltaproteobacteria; Desulfobacterales; Desulfobacteraceae; *Desulfobacter*	*Desulfobacter postgatei*	99%
8928	Bacteroidetes	*Bacteroidetes bacterium*	98%
10330	Proteobacteria; Deltaproteobacteria; Desulfobacterales; Desulfobacteraceae; *Desulfobacter*	*Desulfobacter postgatei*	97%
11521	Proteobacteria; Betaproteobacteria; Hydrogenophilales; Hydrogenophilaceae; *Thiobacillus*	*Thiobacillus* sp.	98%

Supplementary Table S6. Sequences were compared using BLAST against NCBI's non-redundant database.

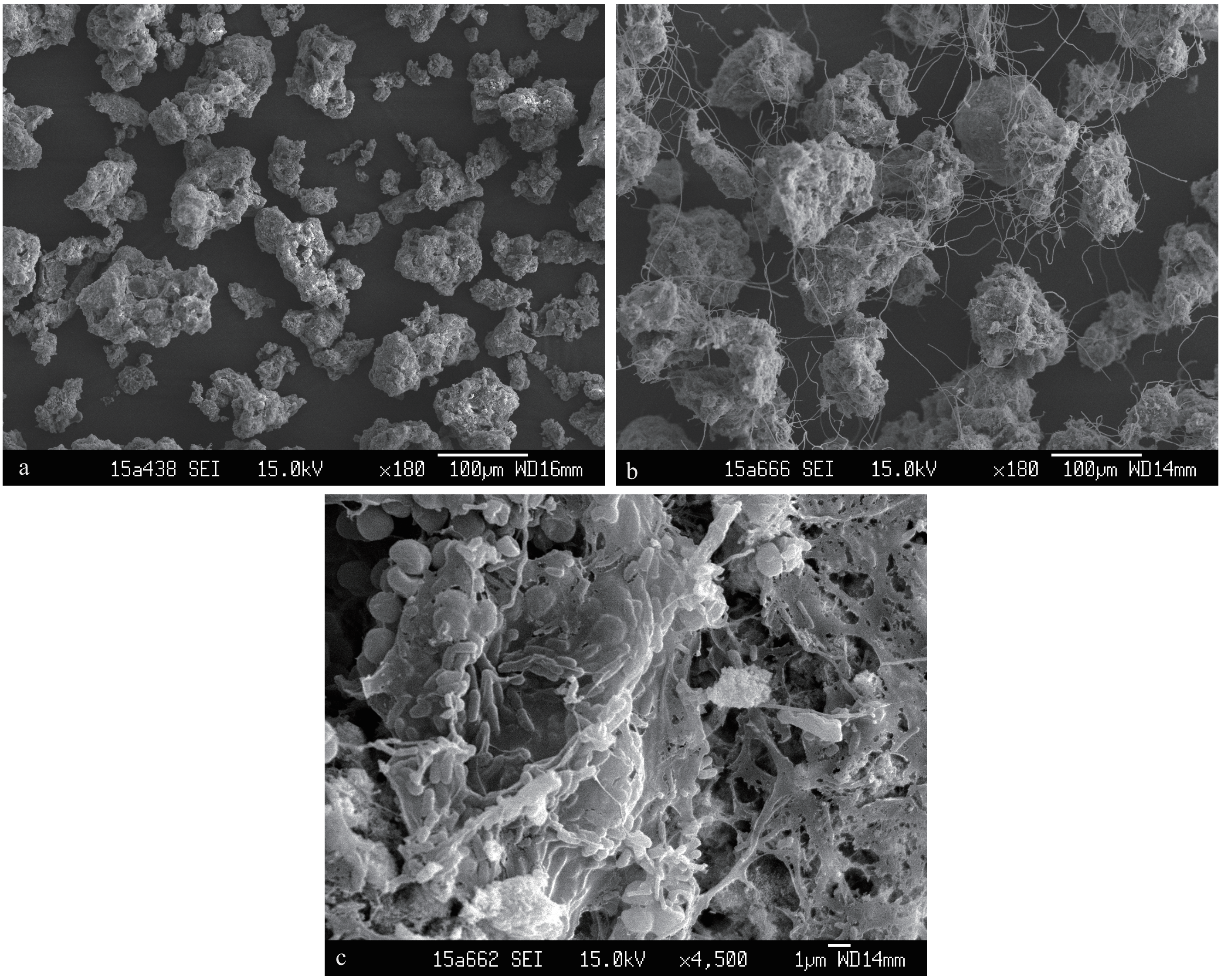

Supplementary Figure S1. Field Emission Scanning Electron Microscope. (a, b) Field Emission Scanning Electron Microscope of S1 and S2 sludge sample at ×180, (c) Field Emission Scanning Electron Microscope of S2 sludge sample at ×4,500.

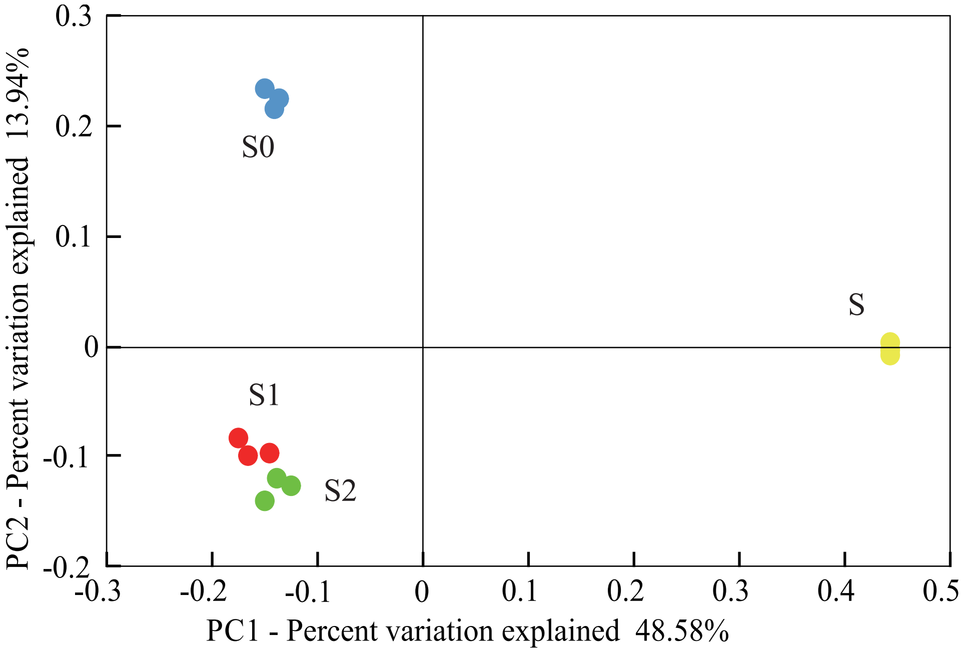

Supplementary Figure S2. Principal Coordinate Analysis (PCoA) of cultured DS-EBPR activated sludge microbial community (S0, S1 and S2), as well as of the inoculum sludge (S) microbial community.

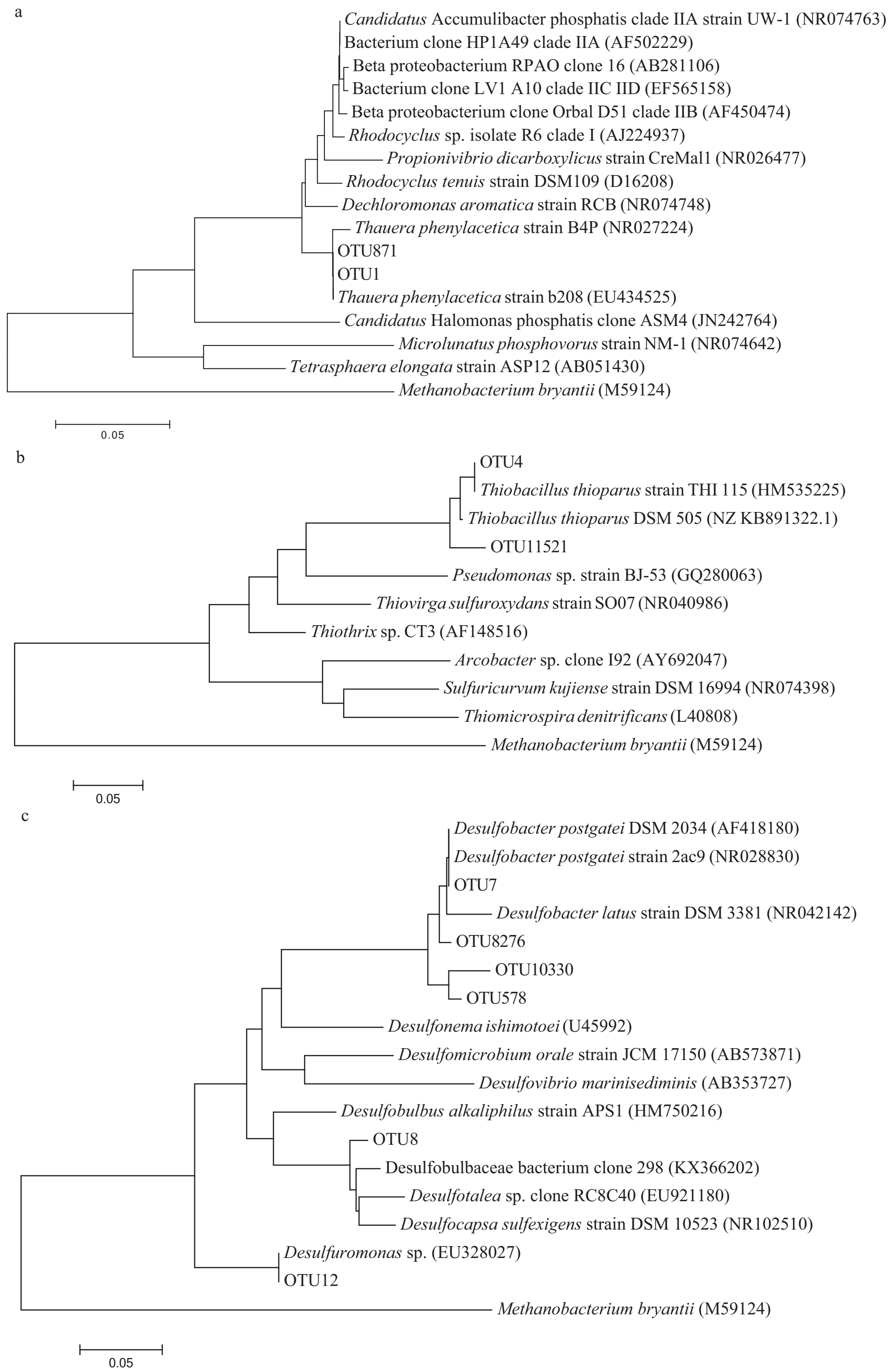

Supplementary Figure S3. Phylogenetic trees of core OTUs. (a) *Candidatus* Accumulibacter phosphatis lineages, (b) Sulfur-oxidizing and sulfide-oxidizing bacterial lineages, and (c) elemental sulfur or sulfate-reducing bacterial lineages, related 16S rRNA genes obtained from the DS-EBPR and some of reported functional bacteria. The neighbor-joining method was used.

References

1. Yu, M. *et al*. Effects of carbon-to-sulfur (C/S) ratio and nitrate (N) dosage on Denitrifying Sulfur cycle-associated Enhanced Biological Phosphorus Removal (DS-EBPR). *Sci Rep*. **6,** 23221 (2016).
2. Barberan, A. *et al*. Why are some microbes more ubiquitous than others? Predicting the habitat breadth of soil bacteria. *Ecol Lett*. **17(7),** 794-802 (2014).
3. Kristiansen, R. *et al*. A metabolic model for members of the genus *Tetrasphaera* involved in enhanced biological phosphorus removal. *ISME J*. **7(3),** 543-554 (2013).
4. Mao, Y., Yu, K., Xia, Y., Chao, Y. & Zhang, T. Genome reconstruction and gene expression of "*Candidatus* Accumulibacter phosphatis" Clade IB performing biological phosphorus removal. *Environ Sci Technol*. **48(17)**, 10363-10371 (2014).
5. Jones, C. M., Stres, B., Rosenquist, M. & Hallin, S. Phylogenetic analysis of nitrite, nitric oxide, and nitrous oxide respiratory enzymes reveal a complex evolutionary history for denitrification. *Mol. Biol. Evol*., **25,** 1955–1966 (2008).
6. Camejo, P. Y. *et al*. *Candidatus* Accumulibacter phosphatis clades enriched under cyclic anaerobic and microaerobic conditions simultaneously use different electron acceptors. *Water Res*. **102,** 125-137 (2016).
7. Zhou, J. *et al*. How sulphate-reducing microorganisms cope with stress: lessons from systems biology. *Nat Rev Microbiol*. **9(6),** 452-466 (2011).
8. Wu, D. *et al*. Simultaneous nitrogen and phosphorus removal in the sulfur cycle-associated Enhanced Biological Phosphorus Removal (EBPR) process. *Water Res*. **49,** 251-264 (2014).
9. Gregersen, L. H., Bryant, D. A. & Frigaard, N. U. Mechanisms and evolution of oxidative sulfur metabolism in green sulfur bacteria. *Front Microbiol*. **2,** 116 (2011).
10. Fike, D. A., Bradley, A. S. & Rose, C. V. Rethinking the Ancient Sulfur Cycle. *Annual Review of Earth and Planetary Sciences*. **43(1)**, 593-622 (2015).
11. Gelder, J. D. *et al*. Monitoring poly (3-hydroxybutyrate) production in cupriavidus necator DSM 428 (H16) with raman spectroscopy. *Anal Chem*. **80(6),** 2155-2160 (2008).
